# Time series analysis of the association between ambient temperature and cerebrovascular morbidity in the elderly in Shanghai, China

**DOI:** 10.1038/srep19052

**Published:** 2016-01-11

**Authors:** Xian-Jing Zhang, Wei-Ping Ma, Nai-Qing Zhao, Xi-Ling Wang

**Affiliations:** 1 Shanghai Insurance Medical Center, Shanghai 200032, People’s Republic of China; 2 Department of Biostatistics, School of Public Health and Key Laboratory of Public Health Safety, Fudan University, Shanghai 200032, People’s Republic of China; 3 Department of Genetics and Genomics Sciences, Icahn School of Medicine at Mount Sinai, New York, NY 10029, US

## Abstract

Research on the association between ambient temperature and cerebrovascular morbidity is scarce in China. In this study, we applied mixed generalized additive model (MGAM) to daily counts of cerebrovascular disease of Shanghai residents aged 65 years or older from 2007–2011, stratified by gender. Weighted daily mean temperature up to lags of one week was smoothed by natural cubic spline, and was added into the model to assess both linear and nonlinear effects of temperature. We found that when the mean temperature increased by 1 °C, the male cases of cerebrovascular disease reduced by 0.95% (95% Confidence Interval (CI): 0.80%, 1.10%) or reduced by 0.34% (95% CI: −0.68, 1.36%) in conditions of temperature was below or above 27 °C. However, for every 1 °C increase in temperature, the female cases of cerebrovascular disease increased by 0.34% (95% CI: −0.26%, 0.94%) or decreased by 0.92% (95% CI: 0.72, 1.11%) in conditions of temperature was below or above 8 °C, respectively. Temperature and cerebrovascular morbidity is negatively associated in Shanghai. MGAM is recommended in assessing the association between environmental hazards and health outcomes in time series studies.

Cerebrovascular disease is a leading cause of disability in adults^1^. Every year, millions of stroke survivors have to adapt to a life with restrictions in activities of daily living as a consequence of cerebrovascular disease. Older age has long been recognized as a risk factor for developing cerebrovascular disease, and the risk goes up significantly for those aged 65 years or older. Men and women tend to have different risks in developing cerebrovascular disease as they differ in socio-economic status, comorbidities and sex steroids^2^,^3^,^4^,^5^. The prevention of cerebrovascular disease is of pivotal importance in maintaining good quality of life in both older men and women.

The incidence of cerebrovascular disease shows a seasonal pattern, and is likely to associate with ambient temperature^6^,^7^. Understanding of the association between transient exposure of temperature and occurrences of cerebrovascular disease might lead to timely implementation of preventive measures and appropriate allocation of medical resources. Although several prior studies have been conducted to evaluate the association of temperature and cerebrovascular morbidity, yet these studies have given conflicting results. For example, Goggins *et al*. found a negative linear association between daily mean temperature and hospital admissions for cerebrovascular diseases in Hong Kong^8^. Low *et al*. suggested a positive association of mean temperature and stroke risk in US^9^. Field *et al*. found no relationship between weather and stroke occurrences in Calgary^10^.

One possible reason for the conflicting results may be that various statistical methods were used to investigate the association of temperature and incidence of cerebrovascular disease. Some studies have investigated the association using generalized linear model (GLM, e.g. Poisson regression)^11^. However, GLM is a set of classic probability-based models assuming independence of observations, therefore GLM might not be appropriate to fit auto-correlated time series data such as incidences of cerebrovascular disease. Generalized additive model (GAM) is another set of probability-based models, and has been commonly adopted in assessing the association between environmental factors and health outcomes of both communicable and non-communicable diseases^12^,^13^. The advantage of GAM over GLM lies in its ability to adjust for both linear and non-linear association of measured and unmeasured confounding factors with health outcomes. However, the selection of appropriate degrees of freedom to adjust for confounders remains controversial. Over-adjustment of confounders may lead to underestimation of true effect, as some variations caused by exposure of interest were absorbed by confounders. Likewise, under-adjustment of confounders could cause spurious association between exposure of interest and health outcome^12^. Therefore, GLM and GAM were relatively simple statistical methods and may fail to take into account the complicating factors including non-linearity, seasonality, autocorrelation and confounding. Recent development of biostatistics suggests that mixed generalized additive model (MGAM) might be a better choice to assess the association between environmental factors and health outcomes, because MGAM includes an autoregressive term as random effect to adjust for autocorrelation of observations and has a robust approach to select appropriate degrees of freedom to adjust for confounders^14^,^15^,^16^.

The primary objective of this study is to assess the association between ambient temperature and cerebrovascular morbidity in the Shanghai residents aged 65 years or older for both men and women. The secondary objective is to compare the performance of GAM and MGAM in assessing the association between temperature and incidence of cerebrovascular disease.

## Methods

### Data

Shanghai is a subtropical city, located in eastern China. It has a total area of 6,341 km^2^ with a total resident population of 14.2 million by the end of 2011^15^. Daily numbers of emergency patients and hospitalized patients diagnosed of cerebrovascular disease in men and women aged 65 years or older from 2007 to 2011 were retrieved from the Official Medicare Database in Shanghai. The official Medicare Database contained medical records in all public hospitals who had Shanghai medical insurance. Two groups of people could have Shanghai medical insurance: residents who have Shanghai household registration or employees who have job contracts longer than six months. The diagnoses of cerebrovascular disease were coded in the International Classification of Diseases, Tenth Revision (ICD10 I60–I69).

Averages of daily mean temperatures measured at all monitoring sites in Shanghai were obtained from the Shanghai Meteorological Bureau. Daily concentrations of particular matters up to 10 micrometers in size (PM_10_), Sulfur dioxide (SO_2_) and nitrogen dioxide (NO_2_) were obtained from the Shanghai Environmental Monitoring Center. Previous studies have indicated that particular matters up to 2.5 micrometers in size (PM_2.5_) was an confounder in assessing the association between temperature and cerebrovascular morbidity^17^. But unfortunately, Shanghai did not start PM_2.5_ surveillance until 2012 that we were unable to adjust for it in the current study. The population sizes from 2007 to 2011 for men and women aged 65 years or older of Shanghai residents were obtained from the Shanghai Research Center on Aging^18^.

### Statistical Analysis

In this study, we used MGAM^19^ to assess the association between ambient temperature and cerebrovascular morbidity in the elderly, stratified by gender. The mathematical representation of MGAM was as follows:


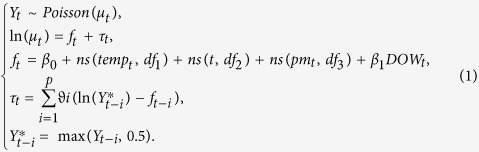
a

*Y*_*t*_ denotes the daily cases of cerebrovascular diseases in the elderly men or women, which was assumed to follow Poisson distribution with mean equaling to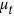. *temp*_*t*_ was the weighted average of daily mean temperature considering the lag effect of temperature in one week, represented as 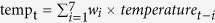 and 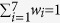. Weighted mean temperature was included into the model using natural cubic spline to account for both linear and non-linear association between temperature and cerebrovascular morbidity. We adjusted for the unmeasured confounding of long term and seasonal trends using natural cubic spline (*ns(t)*), and measured confounding of PM_10_ using natural cubic spline (*ns(pm*_*t*_)) and day of the week as a dummy variable (*DOW*_*t*_) in the model. Daily concentrations of SO_2_ and NO_2_ were not included into the final model because they were almost all statistically insignificant (Table S1). *df*_*1*_, *df*_*2*_ and *df*_*3*_ denote the degrees of freedom (*df*) for temperature, trends and PM_10_, respectively. *df*_*1*_ and *df*_*3*_ was chosen to minimize the Akaike information criterion. *df*_*2*_ was chosen to ensure the seasonal pattern of temperature across years, with detailed methodology described in our previous papers^14^,^15^. 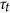 was considered as the random effect to account for the dependence nature of time series data. The coefficients and weights for temperature were estimated by maximum partial likelihood using Newton’s Method.

### Comparison of MGAM and GAM

Although MGAM was believed to outperform the traditional GAM^20^, we repeated the analysis using GAM and compared their performances. The mathematical representation of GAM model was as follows:


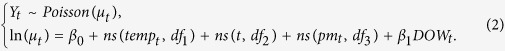


The major difference of MGAM and GAM was that GAM did not have random effect 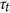 to adjust for the autocorrelation. The performance of GAM and MGAM was assessed by plots of residual autocorrelation function (ACF) and partial autocorrelation function (PACF) considering the lags up to 30 days (around one month). The selected model with better ACF and PACF was adopted to examine the estimated spline for temperature effect on cerebrovascular morbidity in the elderly men and women.

The study was approved by the Institute Review Board (IRB) of the School of Public Health, Fudan University. As aggregated data with no personal information were involved, this study was exempt from obtaining informed consent. All statistical analyses were conducted using R software (version 3.1.2).

## Results

### Descriptive statistics

In Shanghai, there were a total of 146,548 and 181,147 episodes of cerebrovascular diseases for men and women aged 65 years or older from 2007 to 2011. In the elderly men, the number of cerebrovascular diseases decreased from 30,700 to 28,100 from 2007 to 2011, and the corresponding rate decreased from 32.3 to 26.0 per 1,000 population (Table 1). In the elderly women, the number and rate of cerebrovascular disease were 37,000 and 31.9 per 1,000 population in 2007, and increased slightly in 2008, but decreased to 34,800 and 27.4 per 1,000 population in 2011 (Table 1). Women had higher rates of cerebrovascular disease than men, except in 2007 when the rate was 0.4 per 1,000 lower in women than in men (Table 1).

Shanghai had a mild subtropical climate with clear four seasons in a year. The four seasons were usually defined as spring from March–May, summer from June–August, autumn from September–November and winter from December to February. The average of daily mean temperature for the whole study period was around 17 °C, and were 16 °C, 28 °C, 20 °C and 6 °C from spring to winter season. Temperature was negatively correlated with daily numbers of cerebrovascular disease (Spearman correlation=−0.4, *P* < 0.05), with more cases occurred in winter and spring seasons than in summer and autumn seasons.

### Association between temperature and cerebrovascular morbidity

**Figure 1:**
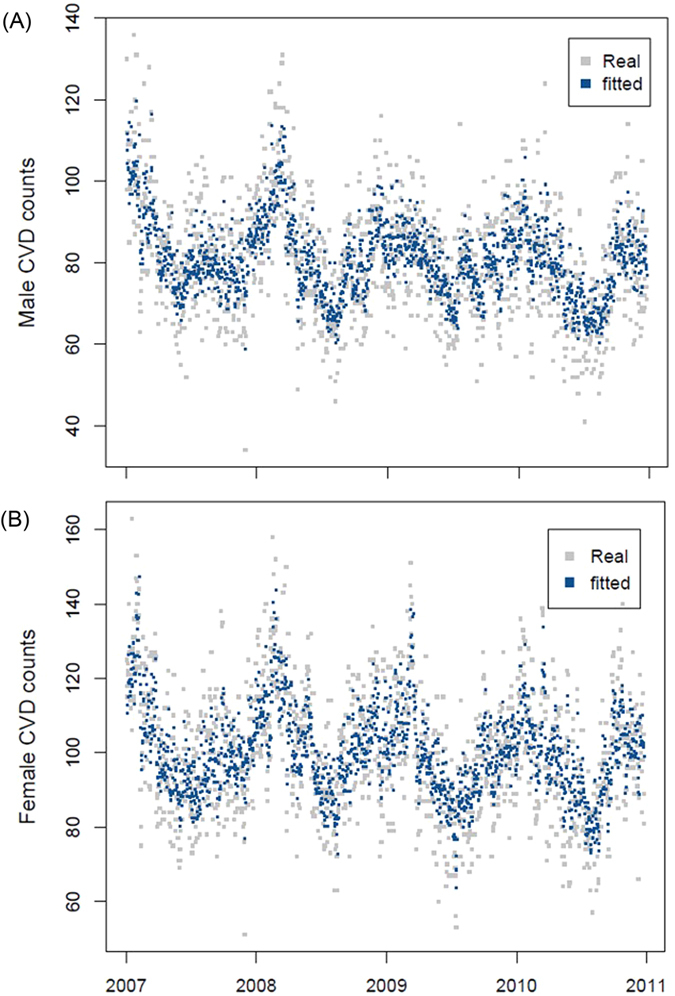
Observed and fitted daily counts of cerebrovascular diseases from MGAM in Shanghai residents aged 65 years or older, 2007–2011. (**A**) Men; (**B**) Women.

**Figure 2:**
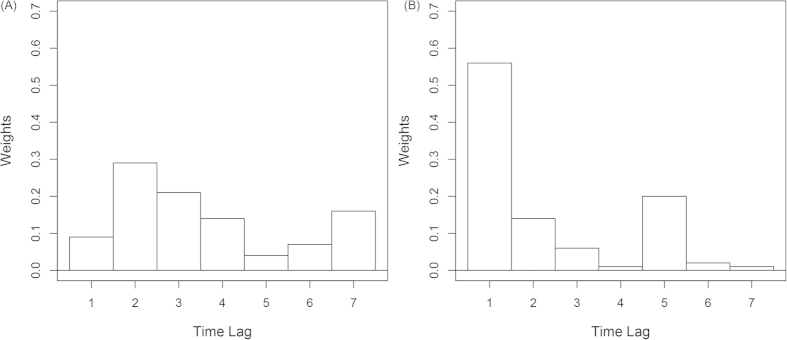
Weights of daily temperature with lags up to one week for males (A) and females (B).

**Figure 3:**
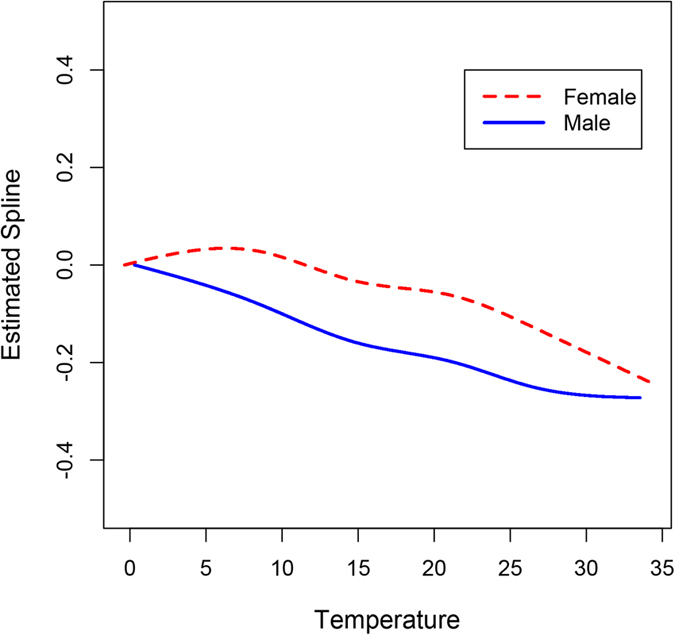
Estimated spline for temperature effect on cerebrovascular morbidity in Shanghai residents aged 65 years or older by gender, 2007–2011.

In general, MGAM well fitted the daily counts of cerebrovascular disease in both elderly men and women (Fig. 1). Temperature had an acute effect on cerebrovascular morbidity in women as the weights of temperature for one day lag was over 0.5 (Fig. 2). However, the weights of temperature were less various in males as all the weights were below 0.3 (Fig. 2). The risk of cerebrovascular disease was negatively associated with temperature (Fig. 3). In the elderly men, the risk of cerebrovascular disease decreased steadily as the temperature increased from the lowest to around 27 °C, but it became stable when the mean temperature exceeded from 27 °C (Fig. 3). By using the 27 °C as the cutoff point for two different linear effects of temperature, when the mean temperature increased 1 °C, the male cases of cerebrovascular disease reduced by 0.95% (95% Confidence Interval (CI): 0.80%, 1.10%) if the temperature was below 27 °C and reduced by 0.34% (95% CI: −0.68, 1.36%) if the temperature was above 27 °C. In the elderly women, the risk of cerebrovascular disease increased slightly as the temperature rose to around 8 °C, and then decreased as the temperature continued increasing (Fig. 3). For every 1 °C increase in temperature, the female cases of cerebrovascular disease increased by 0.34% (95% CI: −0.26%, 0.94%) but decreased by 0.92% (95% CI: 0.72, 1.11%) in conditions of temperature was below or above 8 °C, respectively.

### Performance of GAM and MGAM

**Figure 4:**
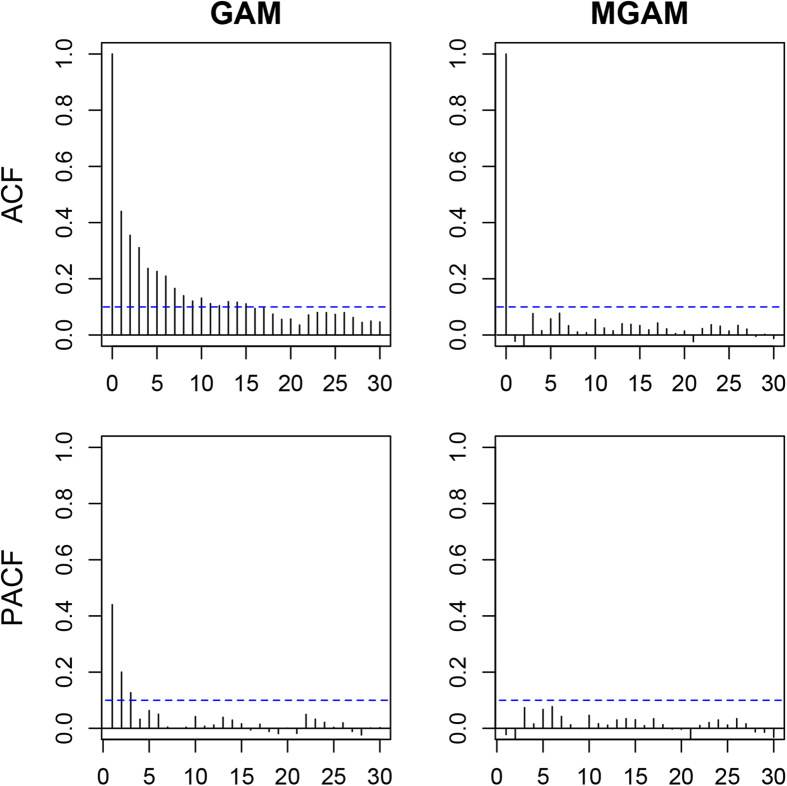
Plots of residual autocorrelation function and partial autocorrelation function of generalized additive model and mixed generalized additive model in the elderly men.

**Figure 5:**
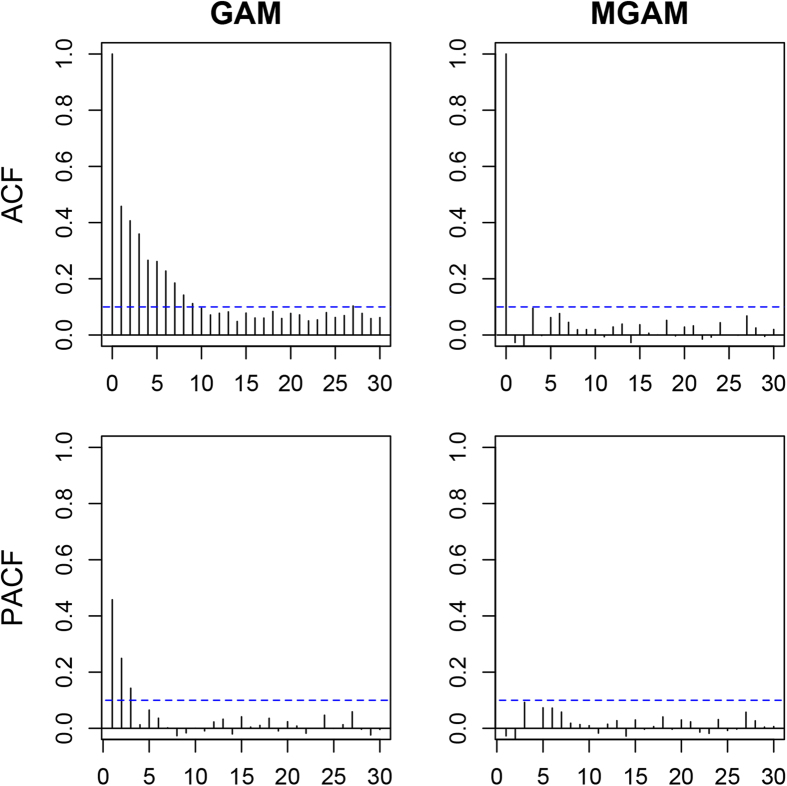
Plots of residual autocorrelation function and partial autocorrelation function of generalized additive model and mixed generalized additive model in the elderly women.

Figures 4 and 5 show the residual ACF and PACF of GAM and MGAM in the elderly men and women, respectively. The residual ACF and PACF of GAM exceeded the boundary of 0.1 for many lags in both elderly men and women, implying the GAM was not appropriate without considering the internal dependence of time series data. In contrast, MGAM performed well as the residual ACF fell within ±0.1 for nonzero lags and the residual PACF fell within ±0.1 for all lags, partially demonstrating the validity of MGAM in this study.

## Discussion

Our study reveals an inverse association between temperature and incidence of cerebrovascular disease. The finding is in line with the findings from Japanese studies, with cerebrovascular morbidity peak occurring in winter/spring months and trough occurring in summer months^21^,^22^. Similarly, studies from Australia, Sweden, Finland and Argentina also reported higher incidences of cerebrovascular disease in winter^23^,^24^,^25^,^26^. An early Framingham study in US did not indicate any seasonal pattern of incidence of total cerebrovascular diseases, but reported a winter peak for cerebral embolism cases^27^. The different proportions of subtypes of cerebrovascular disease might explain the inconsistency of winter peak of total cerebrovascular diseases in Shanghai and US. In 2000, another study compromised of veterans in US demonstrated the association between incidence of cerebrovascular disease and season, which showed the occurrence of ischemic stroke peaking in Mid-May, and bottomed in December^28^. Likewise, the association between temperature and mortality of cerebrovascular disease varied across the world. An epidemiologic study in five cities (Beijing, Tianjin, Shanghai, Wuhan and Guangzhou) in China showed statistical significantly association of cold temperature with cerebrovascular mortality while hot effect was absent^29^. A study in California suggested a negative but not significant association between temperature and cerebrovascular mortality^30^. A study from Russian suggested a U-shape relationship between temperature and cerebrovascular mortality with the temperature threshold set at 18 °C^31^. A study from England also suggested an increased cerebrovascular mortality and elevated ambient temperature^32^. However, our study does not suggest the “heat-effect” of increased cerebrovascular disease morbidity and elevated ambient temperature. The “heat-effect” phenomenon might be explained by the population’s adaption to the environment. Both Russian and England are located at high latitude, so the residents might have already adapted to the cold environment, but very sensitive to the hot weather. Shanghai, on the other hand, is relatively warm, so the residents might not quite sensitive to hot climate, but susceptible to cold weather. Nevertheless, the diverse seasonal pattern of cerebrovascular morbidity and mortality in globe requires further investigation.

Our study found different temperature splines on cerebrovascular morbidity for men and women, particularly in cold climate. The cerebrovascular morbidity decreased in men while surprisingly increased in women when the temperature increased to around 8 °C. The gender difference might be explained by the different social behavior in elderly men and women. The elderly women might prefer staying indoors with air conditions during very cold days, and later exposed to cold weather as the temperature increased a little bit. The elderly men, however, might be not used to the air condition and exposed to the environment regardless of the temperature.

The biological mechanisms for elevated risks of cerebrovascular disease in winter are complex. Low temperature is likely to induce vasoconstriction, resulting to increased blood pressure^33^. Cold exposure may also activate the sympathetic nervous system^34^, with enhanced platelet aggregation. Fibrinogen has been reported to be a risk factor for cerebrovascular disease^35^, and the fibrinogen plasma level was 23% higher in the colder months than in warmer months in the elderly as reported by Crawford^36^. Other biological factors could be increases levels of C-reactive protein in winter months^37^,^38^. All these factors could affect the blood supply to brain in winter and finally caused cerebrovascular disease. A recent study was conducted to investigate the pathophysiologic mechanisms underlying the association of temperature and cerebrovascular hemodynamics based on an elderly cohort, which revealed that temperature was negatively associated with cerebral blood flow velocity and cerebrovascular vasoreactivity and positively associated with cerebrovascular resistence^39^.

Although GAM has been widely accepted in assessing the association between environmental hazards and health outcomes, our study clearly illustrated that GAM could result to inaccurate association if autocorrelation was not appropriately adjust for. MGAM which takes the autocorrelation nature of time series data as random effect, is recommended in future environmental time series modeling.

Our study has several limitations. Firstly, this is an ecologic study which cannot rule out the ecologic fallacy. Individual factors such as socioeconomic status and chronic health conditions cannot be adjusted for in the ecologic study design. Secondly, PM_2.5_ was considered as a confounder in many studying assessing the association between the temperature and cerebrovascular morbidity. But unfortunately, PM_2.5_ cannot be adjusted for in the current study due to the data availability. Third, we could not rule out the disease misclassification for cerebrovascular disease although the rate of misclassification is usually low. Fourth, we did not separate the cerebrovascular disease by subtypes of ischemic stroke or hemorrhagic stroke. We cannot exclude the possibility that the association of cerebrovascular disease and temperature varied by subtypes.

In conclusion, our study suggests a higher cerebrovascular morbidity in cold weather and a lower cerebrovascular morbidity in warm weather. Women tended to have a higher cerebrovascular morbidity than men. For the public health significance, our findings will assist the policy-makers to give appropriate public health education or instructions to people at high risk to develop cerebrovascular disease at different seasons. From the methodology aspect, our study recommends to use MGAM in future environmental time series modeling.

## Additional Information

**How to cite this article**: Zhang, X.-J. *et al*. Time series analysis of the association between ambient temperature and cerebrovascular morbidity in the elderly in Shanghai, China. *Sci. Rep*. **6**, 19052; doi: 10.1038/srep19052 (2016).

## Supplementary Material

Supplementary Information

## Figures and Tables

**Table 1:** Numbers and rates of cerebrovascular diseases in Shanghai residents aged 65 years or older by gender, 2007–2011.

	Males			Females		
	Population (thousands)	Number of cases (thousands)	Rate (per1,000)	Population (thousands)	Number of cases (thousands)	Rate (per 1,000)
2007	950.5	30.7	32.3	1,161.3	37.0	31.9
2008	970.8	30.5	31.4	1,174.2	38.3	32.6
2009	1,005.6	29.0	28.9	1,204.4	35.5	29.5
2010	1,034.5	28.1	27.2	1,230.4	35.5	28.9
2011	1,079.8	28.1	26.0	1,272.4	34.8	27.4
