# Supplementary material for: Time series analysis of the association between ambient temperature and cerebrovascular morbidity in the elderly in Shanghai, China
Source: Sci Rep. 2016 Jan 11;6:19052. doi: 10.1038/srep19052 (PMC4707484; doi:10.1038/srep19052)
Supplement: Supplementary Information [file srep19052-s1.pdf]

Supplementary table for manuscript

Time series analysis of the association between ambient temperature and cerebrovascular morbidity in the elderly in Shanghai, China

Xian-Jing Zhang[1, #], Wei-Ping Ma[2,3, #], Nai-Qing Zhao[2,*], Xi-Ling Wang[2,*]

1. Shanghai Insurance Medical Center, Shanghai 200032, People's Republic of China
2. Department of Biostatistics, School of Public Health and Key Laboratory of Public Health Safety, Fudan University, Shanghai 200032, People's Republic of China
3. Department of Genetics and Genomics Sciences, Icahn School of Medicine at Mount Sinai, New York, NY 10029, US

Table S1. Coefficients of variables from mixed generalized additive models.

	Coefficients for Males				Coefficients for Females			
	Estimate	Std.Error	z value	Pr(>|z|)	Estimate	Std.Error	z value	Pr(>|z|)
(Intercept)	4.67	0.04	105.69	0.00	4.75	0.04	111.77	0.00
time trend component 1	-0.08	0.03	-2.44	0.01	-0.01	0.03	-0.40	0.69
time trend component 2	-0.14	0.04	-3.45	0.00	-0.12	0.04	-2.86	0.00
time trend component 3	-0.10	0.03	-2.95	0.00	-0.06	0.03	-1.74	0.08
time trend component 4	-0.21	0.06	-3.31	0.00	-0.11	0.06	-1.68	0.09
time trend component 5	-0.12	0.03	-3.96	0.00	-0.08	0.03	-2.81	0.00
temperature effect component 1	-0.17	0.03	-5.50	0.00	-0.05	0.03	-1.58	0.11
temperature effect component 2	-0.17	0.04	-4.47	0.00	-0.04	0.03	-1.21	0.23
temperature effect component 3	-0.26	0.03	-8.31	0.00	-0.15	0.03	-5.48	0.00
temperature effect component 4	-0.31	0.07	-4.80	0.00	-0.14	0.06	-2.31	0.02
temperature effect component 5	-0.22	0.04	-5.38	0.00	-0.26	0.04	-6.83	0.00
pm10 effect component 1	-0.03	0.04	-0.74	0.46	-0.10	0.04	-2.68	0.01
pm10 effect component 2	0.01	0.05	0.28	0.78	-0.04	0.05	-0.90	0.37
pm10 effect component 3	0.15	0.07	2.33	0.02	0.03	0.06	0.45	0.65
SO2 effect component 1	0.01	0.03	0.49	0.62	0.02	0.03	0.76	0.45
SO2 effect component 2	0.08	0.05	1.42	0.16	0.01	0.05	0.24	0.81
SO2 effect component 3	0.04	0.06	0.67	0.50	0.02	0.06	0.41	0.68
NO2 effect component 1	0.01	0.02	0.49	0.63	0.00	0.02	0.04	0.97
NO2 effect component 2	-0.03	0.05	-0.59	0.56	0.07	0.05	1.47	0.14
NO2 effect component 3	-0.02	0.05	-0.52	0.60	0.09	0.04	2.22	0.03
Monday	0.07	0.01	8.68	0.00	0.03	0.01	4.64	0.00
Tuesday	-0.03	0.01	-3.00	0.00	-0.07	0.01	-8.88	0.00
Wednesday	-0.03	0.01	-3.69	0.00	-0.06	0.01	-7.00	0.00
Thursday	-0.03	0.01	-3.49	0.00	-0.07	0.01	-8.31	0.00
Friday	-0.04	0.01	-4.35	0.00	-0.06	0.01	-8.17	0.00
Saturday	-0.03	0.01	-3.95	0.00	-0.05	0.01	-6.42	0.00
autoregressive term 1	0.33	0.02	15.83	0.00	0.34	0.02	16.79	0.00
autoregressive term 2	0.22	0.02	10.50	0.00	0.25	0.02	12.39	0.00
